# Oral health-related quality of life of adolescents after orthodontic treatment. A systematic review

**DOI:** 10.4317/jced.55527

**Published:** 2019-02-01

**Authors:** Elena Ferrando-Magraner, Verónica García-Sanz, Carlos Bellot-Arcís, José-María Montiel-Company, José-Manuel Almerich-Silla, Vanessa Paredes-Gallardo

**Affiliations:** 1 Pre-doctoral student, Orthodontic Teaching Unit, Department of Stomatology, Faculty of Medicine and Dentistry, University of Valencia, Spain; 2 Associate lecturer, Orthodontic Teaching Unit, Department of Stomatology, Faculty of Medicine and Dentistry, University of Valencia, Spain; 3 Assistant Lecturer, Orthodontic Teaching Unit, Department of Stomatology, Faculty of Medicine and Dentistry, University of Valencia, Spain; 4 Post-doctoral contract lecturer, Preventive Dentistry Teaching Unit, Department of Stomatology, Faculty of Medicine and Dentistry, University of Valencia, Spain; 5 Tenured lecturer, Preventive Dentistry Teaching Unit, Department of Stomatology, Faculty of Medicine and Dentistry, University of Valencia, Spain; 6 Post-doctoral contract lecturer, Orthodontic Teaching Unit, Department of Stomatology, Faculty of Medicine and Dentistry, University of Valencia, Spain

## Abstract

**Background:**

Given the prevalence of malocclusions and the impact they have on oral health, patients’ quality of life assessments provide useful information, not only in terms of patients’ needs and expectations before treatment, but about whether or not orthodontic treatments meet them satisfactorily. The present systematic review was carried out to evaluate changes in the quality of life of adolescent patients after orthodontic treatment.

**Material and Methods:**

An electronic search was conducted in the Pubmed, Embase, Cochrane and Scopus databases. The review followed PRISMA guidelines for systematic reviews and meta-analyses.

**Results:**

Of the 817 studies identified in the initial search, only 10 met the inclusion criteria. In relation to the instrument used to assess oral health-related quality of life (OHRQoL), half the studies used the oral health impact profile-14 (OHIP-14) and the other half the child perceptions questionnaire (CPQ 11-14). All the studies, with the exception of Benson *et al.*, reported a significant improvement in OHRQoL at the end of treatment.

**Conclusions:**

There is a positive association between OHRQoL and orthodontic treatment in adolescent patients.

** Key words:**Quality of life, life quality, oral health related quality of life, QoL, OHRQoL, orthodontic treatment, adolescents, teenagers.

## Introduction

The main objective of orthodontic therapy is to correct malocclusion. But nowadays, patients’ responses to treatment are more influenced by psychosocial and aesthetic aspects than their oral health status (1). Improvements in both function and aesthetics are supposed to lead to better and more stable psychosocial welfare (2). In this context, it is important for the orthodontist to understand the oral health factors that can affect an individual’s quality of life (QoL), and therefore the relationship between oral health care and the hoped-for improvement in QoL (3,4), known as Oral Health Related Quality Of Life (OHRQoL) (5). OHRQoL indicators will help the clinician assess the patient’s needs and expectations, and support decisions about treatment planning in relation to the individual patient’s concerns (6,7).

There are many questionnaires designed to evaluate OHRQoL but these are subject to a high degree of heterogeneity and most of them are designed to assess adult patients. The “Oral Health Impact Profile” (OHIP) and the “Child Perception Questionnaire 11 to 14 years” (CPQ 11-14) are validated indices, and the most commonly used to assess children and adolescents. The OHIP (Slade y Spencer, 1994) is a self-evaluation tool that analyses patients’ perceptions of the impact of oral disorders on their wellbeing (8). The CPQ 11-14 index was introduced by Jokovic *et al.* (2002) to assess children aged 11-14 years (7). Both of these indices are designed to be completed by the patient (7,9).

Dental malocclusions are a very prevalent disorder among children and adolescents all over the world. The World Health Organization (WHO) places malocclusion in third place in prevalence among all buccodental health problems, following dental caries, and periodontal disease.

When children and adolescents seek orthodontic treatment, this is usually associated with problems of masticatory function, dissatisfaction with their appearance, temporomandibular joint dysfunction, swallowing or speech disorders, susceptibility derived from facial trauma, and/or the possibility of developing caries or periodontal disease (11). Nevertheless, most adolescents seek orthodontic treatment for purely esthetic reasons, a fact that points to an underlying psychosocial factor (12).

Numerous studies have analyzed variations in OHRQoL before, after, and during orthodontic treatment (4,13-17). But most studies suffer important limitations derived from the heterogeneity in patients’ ages (4,14,17), dispersion of the samples’ treatment needs (14,17), poor follow-up, or the fact that the study focuses on only one phase of orthodontic treatment (13,15,16).

Given the prevalence of malocclusion in the general population and its impact on oral health, assessing patient quality of life has great bearing on orthodontic treatment when it comes to determining the needs and expectations of the individual patient and that he/she is satisfied with the treatment received; treatment should lead to an improvement in quality of life (18,19).

The aim of the present study was to conduct a systematic review of all research papers that have studied changes in the QoL of adolescent patients after orthodontic treatment.

## Material and Methods

This systematic review complied with the Preferred Reporting Items for Systematic Reviews and Meta-Analyses (PRISMA) statement (20) and was registered with the PRISMA (PROSPERO) database (reference number CRD42017065093). The research question was: does treatment with fixed orthodontic appliances improve the oral health related quality of life of adolescent patients? 

An initial search was conducted in the Pubmed-Medline, Embase, Cochrane and Scopus databases. A further electronic search for ‘grey literature’ was also made in the New York Academy of Medicine Grey Literature Report. No limits were imposed in terms of publication date or language; the search was updated in May 2017.

A combination of MeSH (Medical Subject Headings) and non-MeSH terms was used to perform the search in the databases, using the following search terms: (adolescent* OR teenager*) AND (orthodontic*) NOT (orthognathic surgery) AND (quality of life, OR life quality, OR oral health related quality of life, OR QoL, OR OHRQoL).

The reference lists of the selected publications were also reviewed manually to identify any further studies that had not been identified in the primary search.

-Study selection criteria:

Two independent reviewers assessed the titles and abstracts of the articles found in electronic searches (E.F-M y V.G-S); in case of any disagreement, a third reviewer was consulted (C.B-A).

The full text was read whenever information provided in the abstract proved insufficient to justify selection/rejection. Afterwards, the full texts of the selected studies were read, registering the reasons for excluding any study at this stage.

The works selected included randomized clinical trials (RCTs), cohort studies, and case-control studies. All papers focused on adolescent patients treated with conventional fixed orthodontic apparatus, whether combined with auxiliary apparatus or not. All studies reported the variable (OHRQoL) both at the start and the end of treatment, assessed by means of validated instruments. Studies with patient samples requiring orthodontic treatment combined with surgery were excluded.

-Data extraction

The following variables were entered in a Microsoft Office Excel 2013 spreadsheet (Microsoft Corporation, Redmond, WA, USA): author, year of publication, study type, sample size, participants lost, demographic variables (age and sex), type of orthodontic treatment, index used to assess OHRQoL, how the questionnaire was filled out, times of assessment, patient follow-up duration, results, and study quality.

-Quality assessment

The quality of the studies was assessed by two independent reviewers (E.F-M and V.P-G) using the Newcastle-Ottawa Quality Assessment Scale (NOS) comprising eight items in three categories. Each item is scored with a star, with the exception of “comparability,” which is scored with two stars, making a maximum score of nine stars. In cases of disagreement between reviewers, the case was discussed and if disagreement persisted, a third reviewer was consulted (C.B-A).

## Results

-Study selection and flow diagram

**Figure 1:**
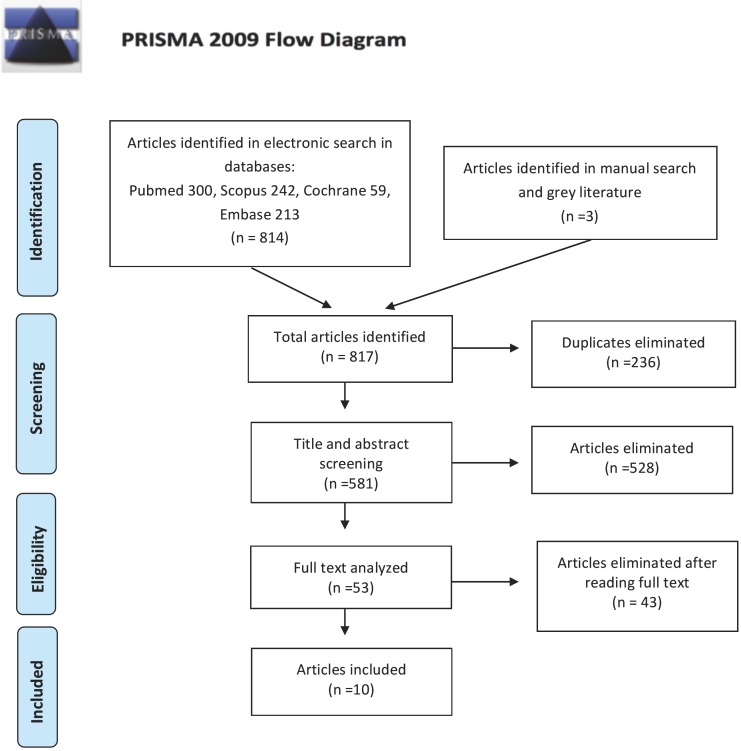
Flow diagram.

The initial electronic search obtained a total of 814 articles (300 in Pubmed-Medline, 242 in Scopus, 213 in Embase, and 59 in Cochrane). The manual search identified a further three articles, and the grey literature search found none. After eliminating duplicates, 581 articles remained. A further 528 articles were rejected after reading the titles and abstracts, leaving a total of 53. Afterwards a detailed analysis of each work, another 43 articles were excluded for the following reasons: failure to meet follow-up criteria (18); failure to meet comparison criteria (6); study did not correspond to the study type specified (4); study did not focus on the age range specified (1); unrelated to the review objectives (14). Finally, ten studies fulfilled the inclusion criteria. The selection process is illustrated in the PRISMA flow diagram (Fig. 1).

-Qualitative synthesis 

The sample sizes of the studies reviewed varied between 27 and 374 patients. All the works focused on adolescents aged between 11 and 18 years, with the exception of two that included patients aged up to 25 years (8,18).

Most of the articles only included treatments with conventional fixed apparatus, although some mentioned additional treatment types (12,21).

Regarding the instrument used to assess OHRQoL, half the articles (8,12,18,22,23) used the Oral Health Impact Profile (OHIP-14) and the other half (1,21,24-26) the Child Perception Questionnaire 11 to 14 years (CPQ 11-14). Most of the works reported that patients filled out the questionnaires without external support.

As for the time when OHRQoL assessments were made, six studies limited assessment to before starting treatment and end of treatment (18,21,24-26); one work assessed OHRQoL before treatment, immediately after treatment and 21 months after bracket debonding (1); and three works performed various assessments throughout treatment (8,22,23). In studies that used the OHIP-14, most (8,12,18,22,23) found the domains undergoing greater changes were related to psychological discomfort and psychological disability. Most of the studies using the CPQ11-14, report greater changes in the domain referring to emotional wellbeing (1,21,25).  Table 1,  Table 1 continue,  Table 1 continue-1,  Table 1 continue-2 summarizes the data collected from the studies reviewed.

**Table 1:**
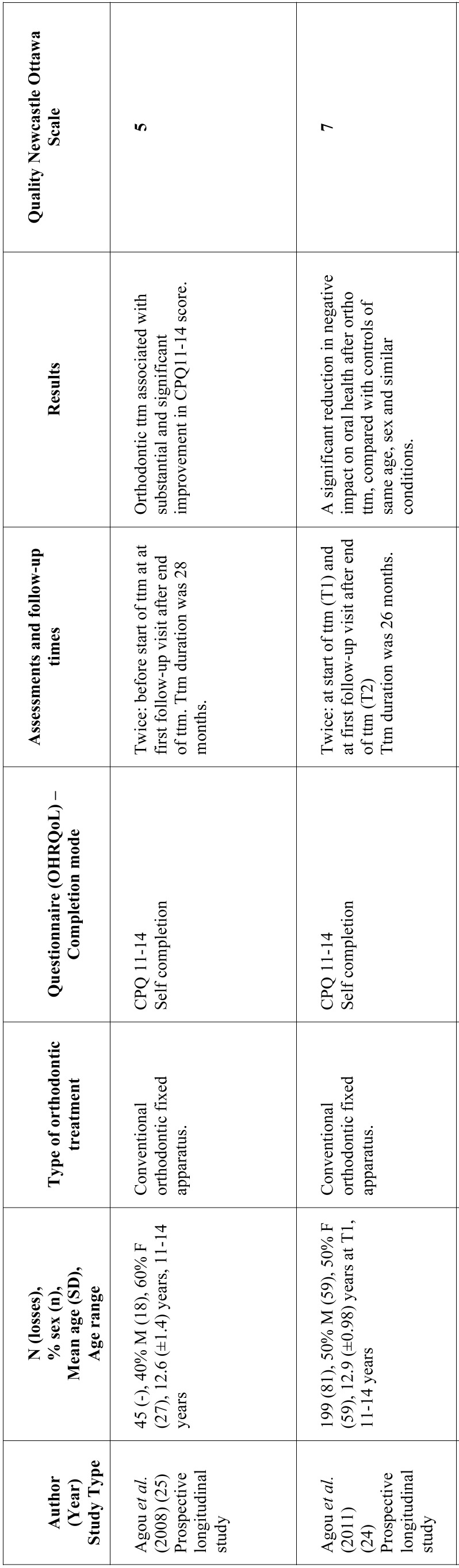
Characteristics of studies investigating the relation between orthodontic treatment and OHRQoL. Approx: approximately; SD: standard deviation; perio dis: periodontal disease; F: feminine; M: masculine; ttm: treatment; ortho ttm: orthodontic treatment.

**Table 1 continue:**
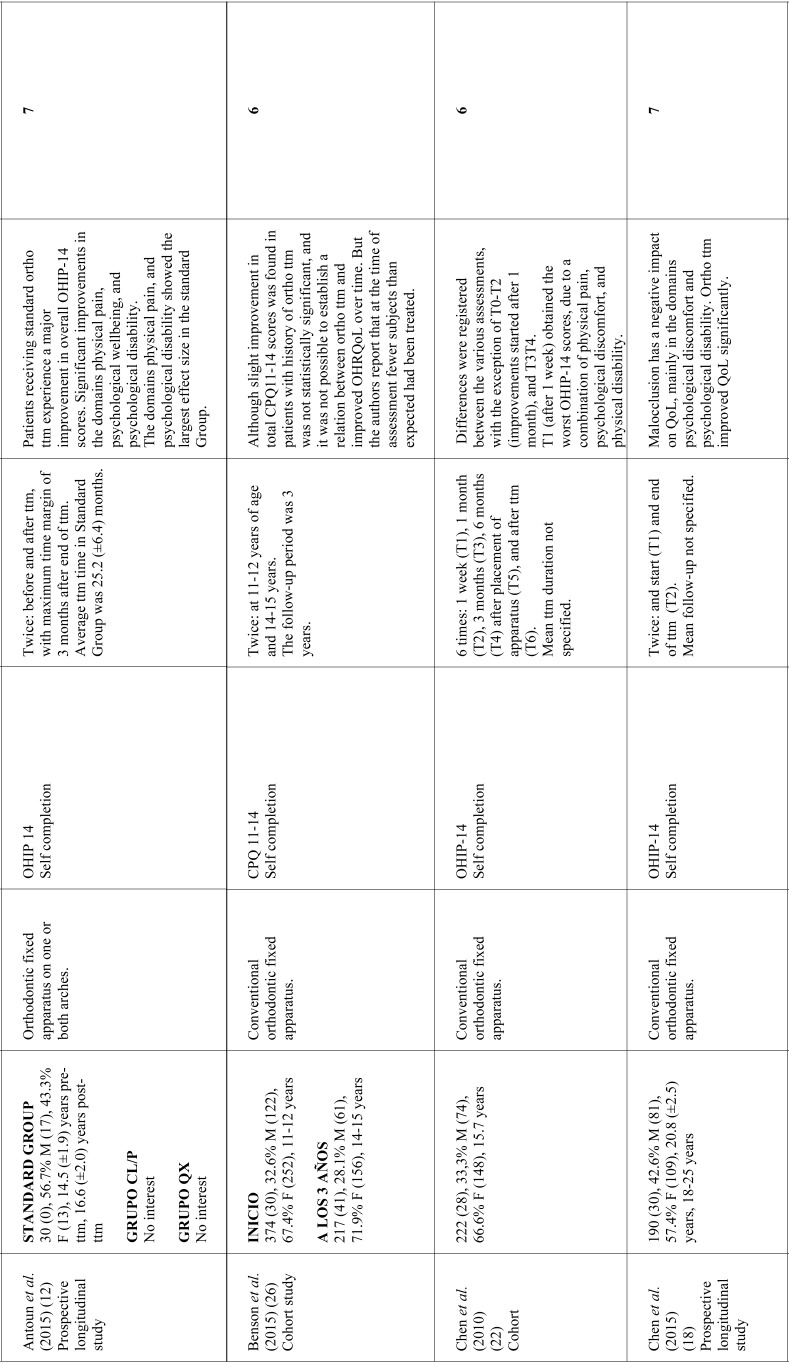
Characteristics of studies investigating the relation between orthodontic treatment and OHRQoL. Approx: approximately; SD: standard deviation; perio dis: periodontal disease; F: feminine; M: masculine; ttm: treatment; ortho ttm: orthodontic treatment.

**Table 1 continue-1:**
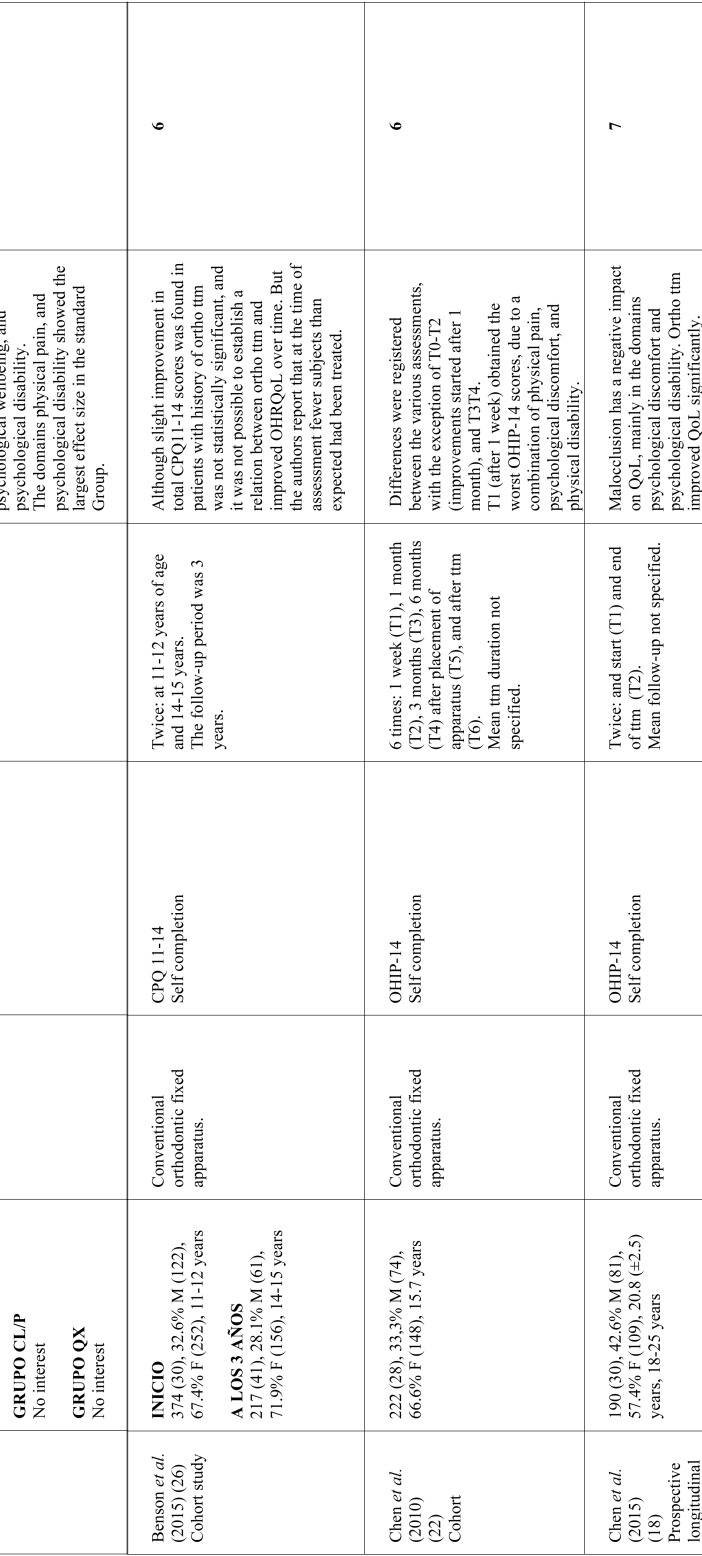
Characteristics of studies investigating the relation between orthodontic treatment and OHRQoL. Approx: approximately; SD: standard deviation; perio dis: periodontal disease; F: feminine; M: masculine; ttm: treatment; ortho ttm: orthodontic treatment.

**Table 1 continue-2:**
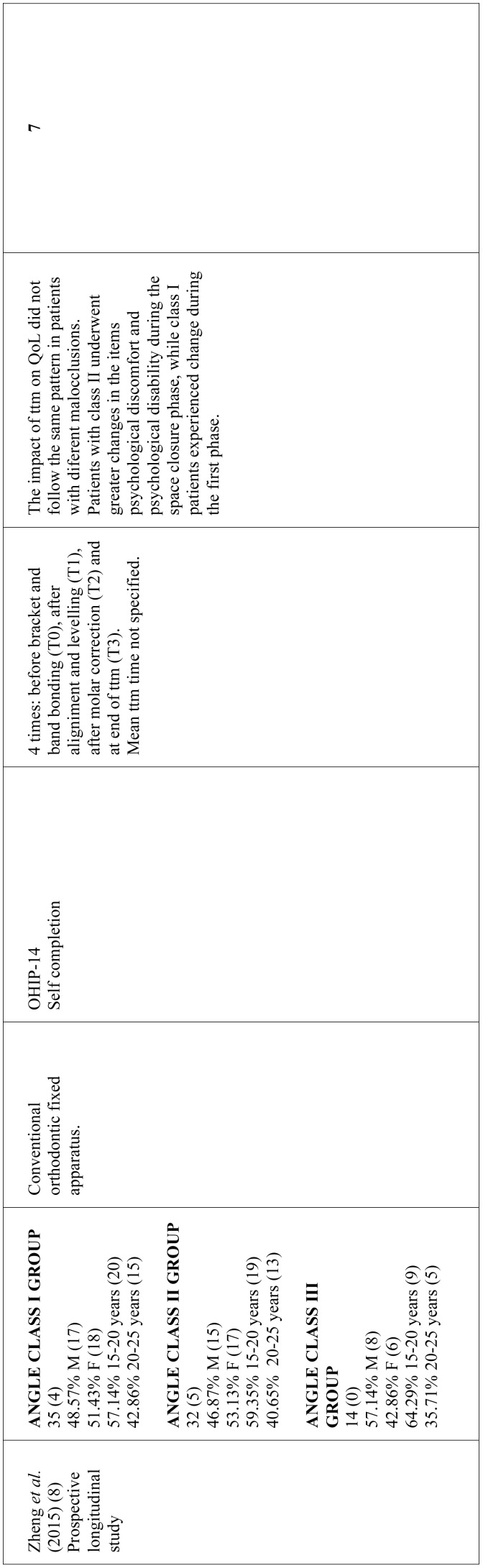
Characteristics of studies investigating the relation between orthodontic treatment and OHRQoL. Approx: approximately; SD: standard deviation; perio dis: periodontal disease; F: feminine; M: masculine; ttm: treatment; ortho ttm: orthodontic treatment.

Most works point to significant differences in OHRQoL between pre- and post-treatment assessments (1,8,12,18,21-25). Among the studies using the OHIP-14, pre- and post-treatment scores varied between 14 and 16 points. In those using the CPQ11-14, values varied between 0.91 and 9.9 points.

None of the studies considered the influence of the type of apparatus employed on QoL. One article emphasized age as a significant factor affecting CPQ 11-14 scores (24).

-Study quality

According to the Newcastle-Ottawa Quality Assessment Scale, all ten studies were considered of moderate quality, none being of high quality, as none of the study designs made it possible to demonstrate that the outcome of interest was not present at the start of the study, and none met the assessment of outcome criterion. The comparability criterion was fulfilled in seven of the studies (1,8,12,18,23,24,26) ( Table 2).

**Table 2:**
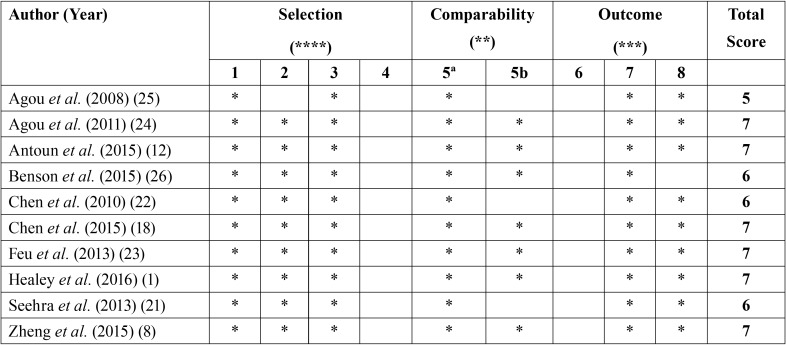
Quality of the studies on the Newcastle-Ottawa Quality Assessment Scale for cohort studies. Criteria: (1) Representativeness of the exposed cohort. (2) Selection of the non-exposed cohort. (3) Ascertainment of exposure. (4) Demonstration that outcome of interest not present at start of study. (5) Comparability of cohorts on the basis of the design or analysis, (5a) for one factor and (5b) for additional factor. (6) Assessment of outcome. (7) Duration of follow-up period. (8) Adequacy of follow-up.

## Discussion

OHRQoL assessment is an essential component in any treatment, and should be performed before any preventative or therapeutic treatment, but especially when treating a malocclusion because of the major psychosocial aspects involved.

The present systematic review set out to analyze current evidence for changes in the OHRQoL of adolescent patients in treatment with orthodontic apparatus between the start of treatment and post-treatment phases. Qualitative analysis of the studies reviewed (1,8,12,18,21-25) concluded that orthodontic treatment by means of fixed apparatus produces a significant improvement in OHRQoL among adolescent patients by the end of treatment, with the exception of one work by Benson *et al.* (26), who did not find any significant differences in pre- and post-treatment OHRQoL.

Although the literature includes various systematic reviews on this topic (27,28), none of them has focused on studies with longitudinal monitoring across the treatment period, so the present review may be considered the first to assess the influence of orthodontic treatment on the QoL of adolescent patients that takes the patients themselves as control subjects, evaluating QoL at the start and end of treatment and eliminating the need for a control group. Most of the studies analyzed did not differentiate between an exposed group and a non-exposed group, but rather a group of subjects undergoing orthodontic treatment with fixed apparatus monitored longitudinally to assess the evolution of QoL (1,8,12,18,21,22,25). Only three of the ten studies reviewed included groups of patients that acted as non-exposed groups (23,24,26).

With regard to the characteristics of the studies reviewed, two specified the apparatus used: Antoun *et al.* (12) used fixed apparatus, while Seehra *et al.* (21) used fixed apparatus alone or in combination with functional apparatus or retainers.

The assessment instruments used in the studies were the OHIP and the CPQ. Chen *et al.* stressed the reliability and validity of the OHIP (22), Antoun *et al.* its simplicity and good discriminatory properties (12) and Zheng *et al.* considered it one of the most sensitive and widely used instruments used for OHRQoL assessment (8). Most of the works using the CPQ11-14 had patient samples that exceeded the questionnaire’ age limits (1,21,26), with the exception of the two studies by Agou *et al.* (24,25).

Among the works that used the OHIP, Zheng *et al.* registered the greatest changes, a reduction of 14.3 points among class III patients (8). As for studies using the CPQ, Agou et al., obtained the greatest change with a reduction of 9.9 points (25).

Limiting QoL assessments to the start and end of treatment could bias the results, and so some studies performed assessments throughout treatment. Zheng et al. assessed QoL four times, finding that class I patients only experienced a significant improvement after the alignment and leveling phase (8). Chen *et al.* applied the questionnaire six times, detecting significant differences between each interval except between the start and the first month, and between three and six months (22). Feu *et al.* assessed OHRQoL at the start of treatment, after one year and after two years, reporting a reduction at each interval, with a more significant reduction at the end of the second year (23).

In relation to the overall results of the studies, Benson *et al.* found a slight improvement in CPQ11-14 scores among patients with a history of orthodontic treatment, although the relationship between the history of orthodontic treatment and the QoL improvement was not statistically significant (26).

The findings of the present systematic review concur with earlier reviews (although these did not apply the same inclusion criteria), which have concluded that improvements in OHRQoL are associated with orthodontic treatment (29-31).

It is important to draw attention to the systematic review and meta-analysis published by Javidi *et al.* (30), as qualitative analysis obtained similar results to the present review, although the earlier review suggested that there were no significant differences between patients who underwent orthodontic treatment and those who did not. However, the work by Javidi *et al.* (30) differed from the present review in that it included both studies with control groups and longitudinal studies.

When it comes to interpreting the results of the present systematic review, certain limitations should be taken into account. Although the study samples were limited to adolescent patients, the age ranges varied from study to study. As for the sex variable, although this was fairly balanced, the percentage of female patients was slightly higher in most of the studies (1,18,22,25,26), which could be due to the fact that the number of women who demand dental treatment is generally higher than the number of men (32). Loss of patients over the course of the study should also be considered a limitation, as the review focused on longitudinal studies with relatively long follow-up periods, which meant that patients were lost in all of them because some moved home (23,24), others did not respond to invitations 

to participate (21), or failed to appear for scheduled appointments (21,26). Nevertheless, only losses of over 40% of the sample by the end of the study period need be considered a limitation.

The application of strict inclusion criteria limited the study to a specific patient group with similar treatment needs. Although the fact that the studies did not all use the same instrument for assessing OHRQoL could be considered a limitation, only two indices were employed (OHIP and CPQ), both being validated instruments which are reproducible, reliable, and adapted to the age ranges studied (8,9,25,33).

To limit publication bias as far as possible, the search strategy was conducted in four databases and complemented with grey literature and manual searches.

The level of evidence of the association under investigation is based on the quality of the studies analyzed, which were considered of moderate quality. The reasons limiting the quality of the studies (according to the criteria applied in the Newcastle-Ottawa Scale) were as follows: no study could demonstrate that the outcome of interest was not already present at the start of the study; being longitudinal studies with long follow-up periods there were considerable losses; none of the studies had randomized samples.

The limited quality and methodology of the studies included in the present systematic review point to the need for further research that analyzes the impact of orthodontic treatment on OHRQoL among adolescents. Studies should have patient samples with clearly defined age ranges, balanced distribution of the sexes, longitudinal follow-up, with losses reduced as far as possible, and using the same validated and reliable assessment instrument.

A positive association was found between OHRQoL and orthodontic treatment in adolescent patients; orthodontic treatment of adolescent patients presenting malocclusion by means of fixed apparatus produces a significantly improved OHRQoL at the end of treatment.
